# The anti-fibrotic effect of inhibition of TGFβ-ALK5 signalling in experimental pulmonary fibrosis in mice is attenuated in the presence of concurrent γ-herpesvirus infection

**DOI:** 10.1242/dmm.019984

**Published:** 2015-09-01

**Authors:** Natalia Smoktunowicz, Robert E. Alexander, Linda Franklin, Andrew E. Williams, Beverley Holman, Paul F. Mercer, Gabor Jarai, Chris J. Scotton, Rachel C. Chambers

**Affiliations:** 1 Centre for Inflammation & Tissue Repair, University College London, London, WC1E 6JF, UK; 2 Institute of Nuclear Medicine, University College London, NW1 2BU, UK; 3 Novartis Institutes of Biomedical Research, Horsham, RH12 5AB, UK

**Keywords:** Pulmonary fibrosis, Viral infection, Collagen, TGFβ, µCT

## Abstract

TGFβ-ALK5 pro-fibrotic signalling and herpesvirus infections have been implicated in the pathogenesis and exacerbation of pulmonary fibrosis. In this study we addressed the role of TGFβ-ALK5 signalling during the progression of fibrosis in a two-hit mouse model of murine γ-herpesvirus 68 (MHV-68) infection on the background of pre-existing bleomycin-induced pulmonary fibrosis. Assessment of total lung collagen levels in combination with *ex vivo* micro-computed tomography (µCT) analysis of whole lungs demonstrated that MHV-68 infection did not enhance lung collagen deposition in this two-hit model but led to a persistent and exacerbated inflammatory response. Moreover, µCT reconstruction and analysis of the two-hit model revealed distinguishing features of diffuse ground-glass opacities and consolidation superimposed on pre-existing fibrosis that were reminiscent of those observed in acute exacerbation of idiopathic pulmonary fibrosis (AE-IPF). Virally-infected murine fibrotic lungs further displayed evidence of extensive inflammatory cell infiltration and increased levels of CCL2, TNFα, IL-1β and IL-10. Blockade of TGFβ-ALK5 signalling attenuated lung collagen accumulation in bleomycin-alone injured mice, but this anti-fibrotic effect was reduced in the presence of concomitant viral infection. In contrast, inhibition of TGFβ-ALK5 signalling in virally-infected fibrotic lungs was associated with reduced inflammatory cell aggregates and increased levels of the antiviral cytokine IFNγ. These data reveal newly identified intricacies for the TGFβ-ALK5 signalling axis in experimental lung fibrosis, with different outcomes in response to ALK5 inhibition depending on the presence of viral infection. These findings raise important considerations for the targeting of TGFβ signalling responses in the context of pulmonary fibrosis.

## Abstract (precis)

**Summary:** This article describes newly identified intricacies for the TGFβ-ALK5 signalling axis in experimental lung fibrosis and reports different outcomes in response to ALK5 inhibition depending on the presence of viral infection. These findings raise important considerations for the future targeting of TGFβ signalling responses in the context of pulmonary fibrosis.

## INTRODUCTION

Idiopathic pulmonary fibrosis (IPF) is the most progressive and fatal of all fibrotic conditions, with a median survival of 3 years. The pathomechanisms involved remain poorly understood, but current hypotheses propose that this condition arises as a result of repetitive epithelial injury followed by a highly aberrant wound healing response in genetically susceptible and aged individuals (reviewed in Datta et al., 2011). The classical histopathological pattern of IPF presents as usual interstitial pneumonia (UIP), with evidence of patchy epithelial damage and hyperplasia combined with abnormal proliferation of mesenchymal cells, concomitant with overproduction and disorganized deposition of extracellular matrix (ECM). Fibrotic foci, the histopathological hallmark of UIP/IPF, comprise accumulations of fibroblasts and myofibroblasts within an extensive ECM underlying injured and reparative epithelium, and are widely considered to represent the leading edge of the fibrotic response.

Although the aetiology of IPF remains unknown, studies examining the role of infection in IPF implicate viral infections, especially human herpesviruses (HHVs), as important contributors to the initiation and progression of this condition (reviewed in Molyneaux and Maher, 2013). Current evidence, albeit from small IPF cohort studies, suggests a role for Epstein-Barr virus (EBV), human cytomegalovirus (HCMV), human herpesvirus-8 (HHV-8) and human herpesvirus-7 (HHV-7) in the progression of fibrosis (Calabrese et al., 2013; Egan et al., 1995; Tang et al., 2003; Vergnon et al., 1984). Moreover, there is evidence linking viral infection with the incidence of acute exacerbation of IPF (AE-IPF) (Wootton et al., 2011), a life-threatening complication that presents as worsening of dyspnoea and an accelerated decline in lung function (Collard et al., 2007).

Animal models of fibrosis have established a causal role for viral infection in the progression of experimental pulmonary fibrosis (Ashley et al., 2014; McMillan et al., 2008; Mora et al., 2005; Vannella et al., 2010). Murine γ-herpesvirus 68 (MHV-68) is closely related to EBV and, like its human counterpart, infects the respiratory epithelium and establishes life-long latency in the host (Nash et al., 2001). This viral tropism for alveolar epithelial cells II (AEC II) contributes to dysregulated epithelial repair and surfactant abnormalities associated with increased apoptosis and alveolar collapse (Lawson et al., 2008). Moreover, latent viral infection alters the phenotype of infected alveolar epithelial cells and fibroblasts, leading to increased TGFβ production and activation (Stoolman et al., 2010; Vannella et al., 2010), as well as increased fibroblast responsiveness to this cytokine, especially in aged mice (Naik et al., 2011).

Current evidence supports a central role for TGFβ in the pathogenesis of fibrosis, including human and murine pulmonary fibrosis (Santana et al., 1995). TGFβ is a potent promoter of extracellular matrix production and promotes fibroblast-to-myofibroblast differentiation, as well as epithelial cell apoptosis (reviewed in Fernandez and Eickelberg, 2012). Multiple approaches that disrupt either TGFβ activation or signalling through direct cytokine inhibition (Giri et al., 1993), Smad3 knockout (Bonniaud et al., 2004), TGF-βRII receptor knockout (Li et al., 2011), integrin αvβ6 knockout (Koth et al., 2007; Morris et al., 2003) or antibody neutralization (Horan et al., 2008) have offered protection in experimental models of pulmonary fibrosis.

**TRANSLATIONAL IMPACT**

**Clinical issue**

Idiopathic pulmonary fibrosis (IPF) is the most progressive and devastating form of lung fibrosis, with a median survival of less than 3 years. Episodes of rapid deterioration, termed acute exacerbations (AE), occur in roughly 10% of individuals with IPF annually, and are a leading cause of morbidity and mortality. Key features of AE include new pulmonary changes and abnormalities that are visible on high-resolution computerized tomography (HRCT) scans; notably, new bilateral ground-glass abnormalities and consolidation superimposed on a background IPF. Histological evaluation of AE-IPF reveals interstitial oedema, hyaline membrane formation and extensive diffuse alveolar damage and haemorrhage. Although the aetiology and mechanisms of AE-IPF are not fully elucidated, current evidence links infection with herpesviruses to the rapid progression of pulmonary fibrosis. TGFβ has been widely implicated in the pathogenesis of pulmonary fibrosis, and strategies that interfere with excessive TGFβ signalling or activation are currently a major focus of therapeutic drug development in this and other fibrotic conditions. The aim of this study was to employ novel micro-computed tomography (μCT) imaging in combination with standard fibrotic end points in order to fully characterize the pathophysiological responses in a two-hit model of murine γ-herpesvirus 68 (MHV-68) infection on the background of experimentally induced pulmonary fibrosis, and to test the therapeutic effect of blocking TGFβ-ALK5 signalling in the presence of concomitant viral infection.

**Results**

This study revealed that concomitant viral infection on the background of pre-existing bleomycin-induced fibrosis in mice leads to prominent and extensive inflammatory changes that are reminiscent of ground-glass opacities and consolidation reported in individuals with AE-IPF. Blocking TGFβ-ALK5 signalling by therapeutic dosing with the potent and selective ALK5 antagonist SB525334 was highly effective in blocking the progression of fibrosis in the single-hit bleomycin-alone injured mouse model, but the anti-fibrotic effect of this agent was dramatically reduced in the presence of concomitant viral infection. In contrast, this inhibitor was highly effective in attenuating extensive inflammatory cell infiltration associated with concomitant viral infection and it enhanced the antiviral cytokine response.

**Implications and future directions**

These studies highlight the pleiotropic nature of the TGFβ-ALK5 signalling axis in pulmonary fibrosis, with different outcomes in response to ALK5 inhibition depending on the presence of viral infection. These findings thus raise important considerations for the future targeting of TGFβ signalling in the context of pulmonary fibrosis: different outcomes on fibrotic progression are expected in stable IPF versus AE-IPF associated with viral infection.

The aim of this study was to further our understanding of the mechanistic links between MHV-68 infection, TGFβ signalling and lung fibrosis. We first established and characterized a model of MHV-68 infection on the background of bleomycin-induced pulmonary fibrosis using standard endpoints (analysis of total lung hydroxyproline) and further investigated disease pathophysiology using *ex vivo* micro-computed tomography (µCT) scanning of whole lungs (Scotton et al., 2013). To investigate the potential role of TGFβ signalling in this model, we employed a highly selective, ATP-competitive activin receptor-like kinase 5 (ALK5; also known as TGF-βRI) inhibitor, SB525334, which has a proven therapeutic effect in single-hit models of experimental pulmonary fibrosis (Bonniaud et al., 2005; Scotton et al., 2013). Taken together, our data reveal previously unknown intricacies for the TGFβ signalling axis in experimental lung fibrosis, with different outcomes observed in response to ALK5 inhibition depending on the presence or absence of viral infection. These findings raise potential clinical considerations for the future targeting of the TGFβ pathway in the context of pulmonary fibrosis, including IPF.

## RESULTS

### Two-hit model of MHV-68 infection on the background of bleomycin-induced fibrosis

In order to establish a two-hit model of MHV-68 infection on the background of fibrosis, mice were challenged with an oropharyngeal instillation of bleomycin (25 IU/mouse) on day 0, followed by an intranasal infection with MHV-68 (1×10^5^ PFU) on day 14. To further our understanding of the mechanistic links between MHV-68 infection and TGFβ signalling in this model, the ALK5 inhibitor (SB525334) was administered therapeutically from day 15 after bleomycin injury through to the end of the experiment at day 28.

**Fig. 1.**
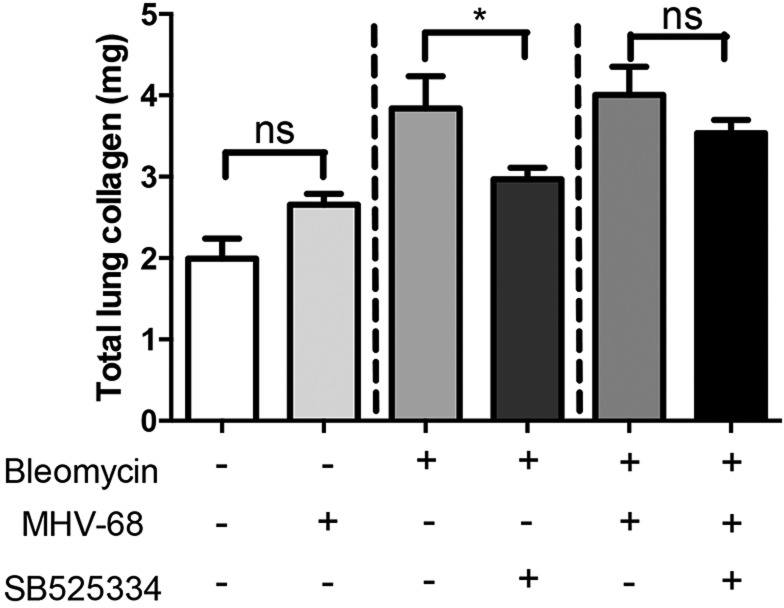
**The anti-fibrotic effect of TGFβ-ALK5 signalling inhibition is attenuated in the two-hit model of MHV-68 infection on the background of pre-existing fibrosis.** Total lung collagen was quantified by reverse-phase HPLC 28 days post-oropharyngeal bleomycin instillation (corresponding to 14 days p.i. with MHV-68). The ALK5 inhibitor SB525334 was administered according to a therapeutic dosing regimen during the progressive fibrotic phase (from day 15 post-bleomycin-instillation; corresponding to 1 day p.i.). MHV-68 infection in saline control lung did not significantly increase lung collagen levels. SB525334 attenuated lung collagen accumulation in the single-hit model of lung fibrosis, but the anti-fibrotic effect of SB525334 was attenuated in the two-hit model of fibrosis with concomitant infection of fibrotic lung. Data are representative of mean±s.e.m., *n*=3 for saline groups and *n*=8 for bleomycin groups; statistical analysis, Student’s *t*-test, *P<0.05.

Total lung collagen was measured by quantifying hydroxyproline levels by reverse-phase high performance liquid chromatography (HPLC) (Fig. 1). MHV-68 infection alone [saline (Sal)+MHV68] had no significant effect on total lung collagen levels when compared to uninfected control lungs (Sal). Administration of bleomycin (Bleo) resulted in a doubling of lung collagen deposition, which was significantly attenuated by SB525334 treatment in the Bleo+SB525334 group (mean±s.e.m. of Bleo vs Bleo+SB525334, 3.8±0.4 mg vs 2.97±0.14 mg, *P*=0.04). MHV-68 infection on the background of existing lung fibrosis (Bleo+MHV-68) did not increase total lung collagen levels compared to the Bleo group. Interestingly, in the two-hit model, there was no difference in total lung collagen between the Bleo+MHV-68+SB525334 group compared with the Bleo+MHV-68 group (mean±s.e.m. of Bleo+MHV-68 vs Bleo+MHV-68+SB525334, 4±1 mg vs 3.5±0.4 mg, *P*=0.6). These observations were further confirmed by the Sircol assay (supplementary material Fig. S1). Taken together, these data led us to conclude that SB525334 attenuates fibrosis in the single-hit model but that the therapeutic effect of this inhibitor is largely lost in the two-hit model.

### µCT characterization of the two-hit model

**Fig. 2.**
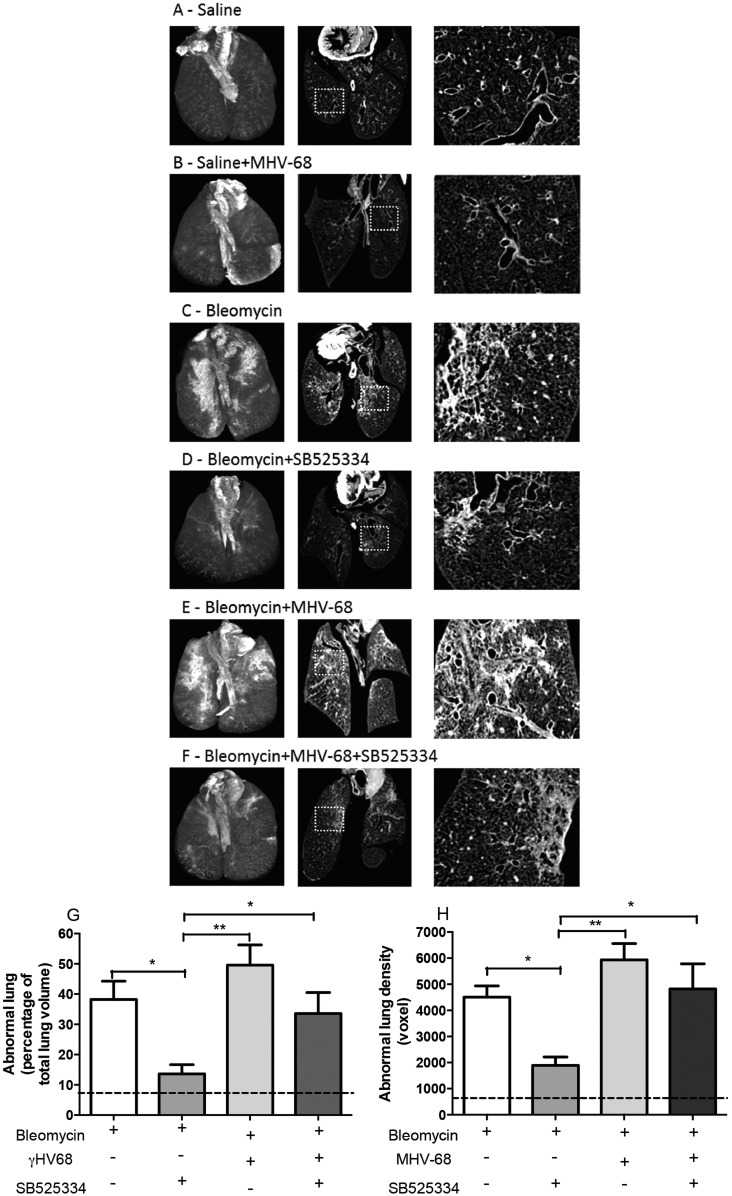
**μCT characterization and quantification of the pathological changes in the single- and two-hit models.** 3D volume reconstruction (left panels, dorsal view) and representative coronal µCT sections (middle panels) with higher (4×) magnification of the highlighted insert (right panel). Mice treated with saline (Sal; A) or Sal+MHV-68 (B) show normal lung morphology; Bleomycin (Bleo)-treated mice (C) show dense subpleural fibrotic lesions, which are attenuated in the Bleo+SB525334 group (D); Bleo+MHV-68 mice (E) show evidence of extensive ground-glass opacities radiating from airways and overlying areas of dense consolidation; Bleo+MHV-68+SB525334 mice (F) reveal dense consolidation with reduced areas of ground-glass opacities. InForm analysis demonstrates an increase in the percentage of abnormal lung area (G) and density (H) in Bleo lungs above the Sal control (dotted line). No significant difference was observed between the Bleo and Bleo+MHV-68 groups. Administration of SB525334 from day 15 post-bleomycin-instillation (and 1 day p.i.) significantly attenuated lung pathology in the Bleo group but not in the two-hit Bleo+MHV-68 model. The data are representative of mean±s.e.m., one-way ANOVA, *P<0.05, **P<0.01, comparison of all Bleo-challenged groups (*n*=5).

*Ex vivo* µCT was subsequently used to further investigate the effect of SB525334 treatment in this two-hit model. Fig. 2 shows representative 3D volume reconstructions (left panels) with corresponding mid-lung coronal µCT sections (middle panels) and magnification of key pathological changes (right panels) for lungs at day 28. Sal+MHV-68 lungs were indistinguishable from Sal control lungs, with both groups displaying an equally homogenous appearance with a network of airways in a virtually transparent parenchyma (Fig. 2A,B). In the Bleo group, peripheral dense fibrotic lesions were clearly visible, particularly on the dorsal side of the lungs (Fig. 2C), as previously reported for oropharyngeal bleomycin instillation (Lakatos et al., 2006; Scotton et al., 2013). Coronal sections revealed prominent sub-pleural scarring, interlobular septal thickening and traction bronchiectasis (Fig. 2C). In Bleo+SB525334 lungs, scarring and fibrosis were noticeably reduced (Fig. 2D). In contrast, in the two-hit model, lungs displayed extensive areas of dense consolidation with overlapping diffuse ground-glass opacities indicative of inflammatory changes concentrated around the airways (Fig. 2E). SB525334 treatment visibly reduced the ground-glass appearance in the two-hit model but the fibrotic lesions remained largely unaffected (Fig. 2F).

### Quantification of changes observed in µCT scans

The abnormal lung area and lung density were subsequently quantified using tissue segmentation analysis of the whole lungs. Bleomycin injury alone resulted in ∼40% of the total lung volume being characterized as abnormal; this was reduced to ∼15% following SB525334 treatment (Fig. 2G). In the Bleo+MHV-68 group, ∼50% of the lung volume was categorized as abnormal. SB525334 treatment in this two-hit model only showed a modest therapeutic effect. Analysis of lung density revealed that total lung density was increased by fourfold for the Bleo group compared with the Sal group (Fig. 2H). This increase was reduced by ∼50% in the Bleo+SB525334 group, confirming the beneficial therapeutic effect of ALK5 inhibition in the single-hit model (Fig. 2H). The voxel density score was highest for the Bleo+MHV-68 two-hit lungs and this was not significantly reduced in the Bleo+MHV-68+SB525334 group.

**Fig. 3.**
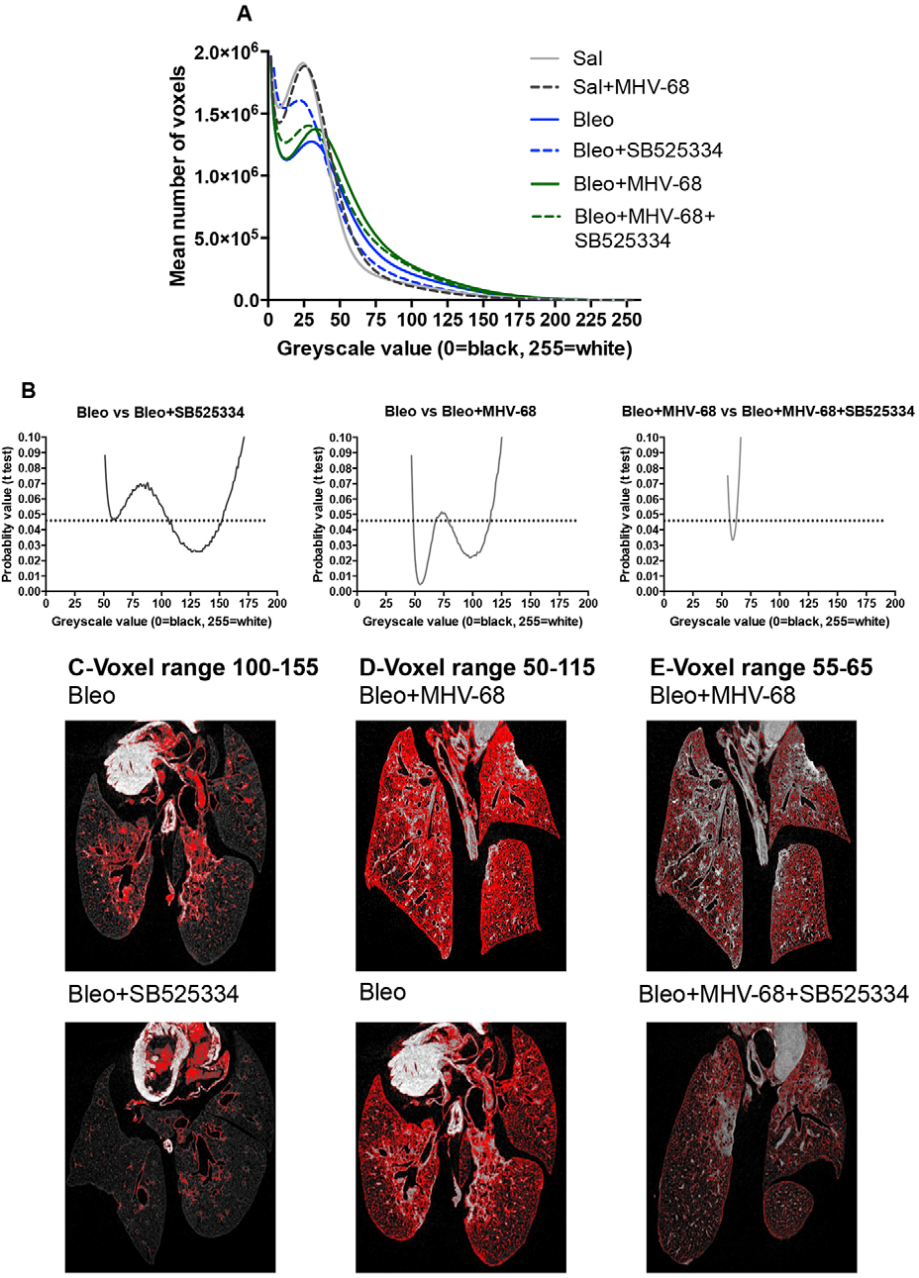
**Voxel density distribution analysis shows key differences between the single- and two-hit models.** Density distribution histograms show the mean number of voxels plotted against greyscale density values (0=air/black, 255=dense tissue/white) for each experimental group. (A) A shift towards higher voxel densities is observed for bleomycin (Bleo)-treated lungs due to Bleo-induced injury, and a further shift to the right for the two-hit Bleo+MHV-68 group is indicative of additional injury. This density shift is reduced in the Bleo+SB525334 group, suggestive of attenuated fibrosis but not in Bleo+MHV-68+SB525334 lungs. (B) The differences in proportion of density voxels for each greyscale bin between Bleo-instilled groups (*n*=5 animals per group), which account for the shifts in the histograms, were analyzed by Student’s *t*-test at each bin. The data are shown as graphs of probability (*y*-axis) versus greyscale density value (*x*-axis), with the significance cut-off set at 0.05 (indicated by the dotted line). (C-E) Distribution of significantly different voxel densities was visualized on representative µCT scans (red pixels): (C) Bleo and Bleo+SB525334 show voxel localization to fibrotic lesions; (D) Bleo+MHV-68 and Bleo lungs show voxel distribution in fibrotic lesions and dispersed throughout the parenchyma; (E) Bleo+MHV-68 and Bleo+MHV-68+SB525334 lungs show voxel distribution dispersed throughout the parenchyma.

In order to further investigate differences in lung morphology, we next performed voxel density distribution analysis for each lung based on the unsegmented µCT data. The mean number of voxels per lung at each greyscale density value (0-255) was calculated and plotted as a histogram (Fig. 3A). Statistically significant differences in the density voxel distribution between individual experimental groups were evaluated by a probability *t*-test (Fig. 3B). A clear separation in the density distribution was observed between Sal, Bleo and Bleo+MHV-68 lungs, with a marked shift towards higher density voxels in the Bleo group, which was further increased in the Bleo+MHV-68 group.

Significantly different voxel distributions between the Bleo and Bleo+SB525334 groups fell in the greyscale density range of 100-155; these voxels localized to fibrotic lesions in the Bleo group (Fig. 3C). Significant increases over a wide voxel range (50-115) were observed in the Bleo+MHV-68 group compared with Bleo (Fig. 3D). In the Bleo+MHV-68 lungs, these voxels corresponded to extensive areas of parenchyma with diffuse ground-glass opacities, in addition to fibrotic lesions. Treatment with SB525334 had a significant effect on a very narrow range of voxels (50-65) in the two-hit group, again indicating the lack of therapeutic effect of ALK5 inhibition on fibrosis in the two-hit model (Fig. 3E).

### Quantification of inflammation in the two-hit model

**Fig. 4.**
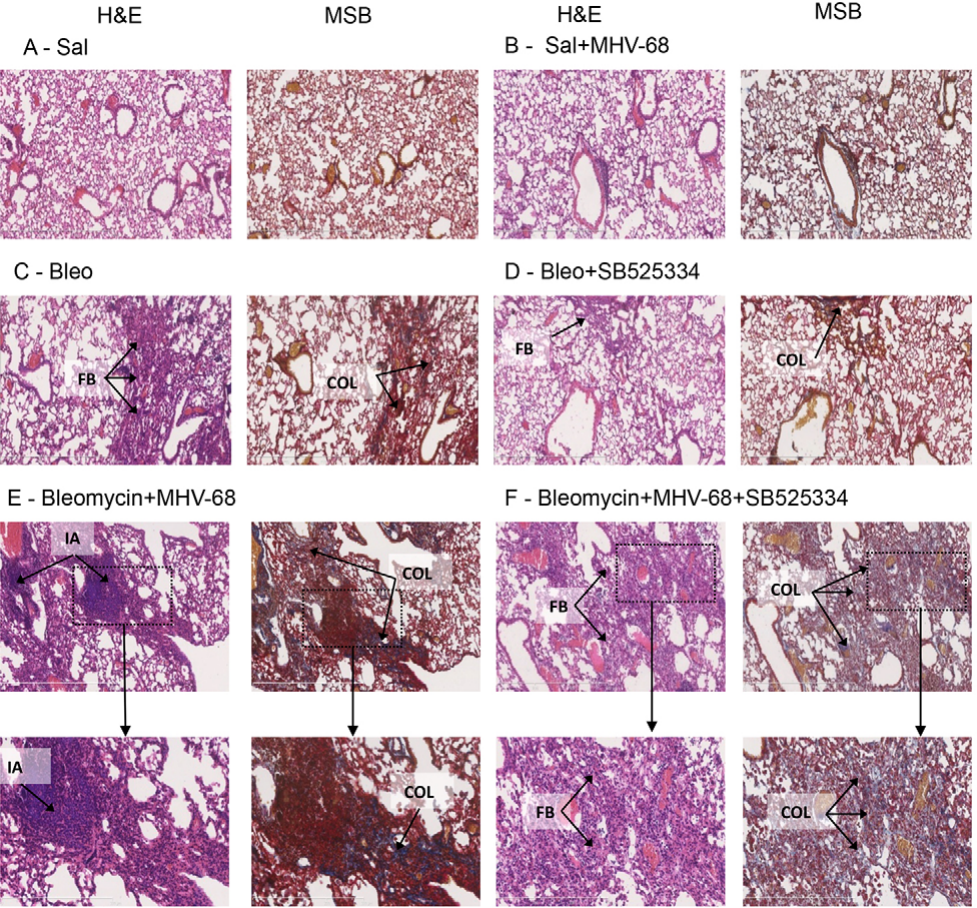
**Histological analysis of the single- and two-hit models.** Representative H&E and MSB sections (100× original magnification; scale bars: 600 μm) are shown for each experimental group as annotated. Saline (Sal) and Saline+MHV-68 lungs show normal morphology (A,B); (C) typical examples of the fibrotic lesions with evidence of collagen deposition observed in the Bleo group. These are greatly reduced in Bleo+SB525334 lungs (D); Bleo+MHV-68 lungs show extensive fibrotic lesions with collagen deposition and dense inflammatory cell aggregates that appear less frequent and more dispersed in Bleo+MHV-68+SB525334 lungs (E,F, boxed area is 200× magnification; scale bars: =300 μm). FB, fibrosis; COL, collagen; IA, inflammatory cell aggregates.

**Fig. 5.**
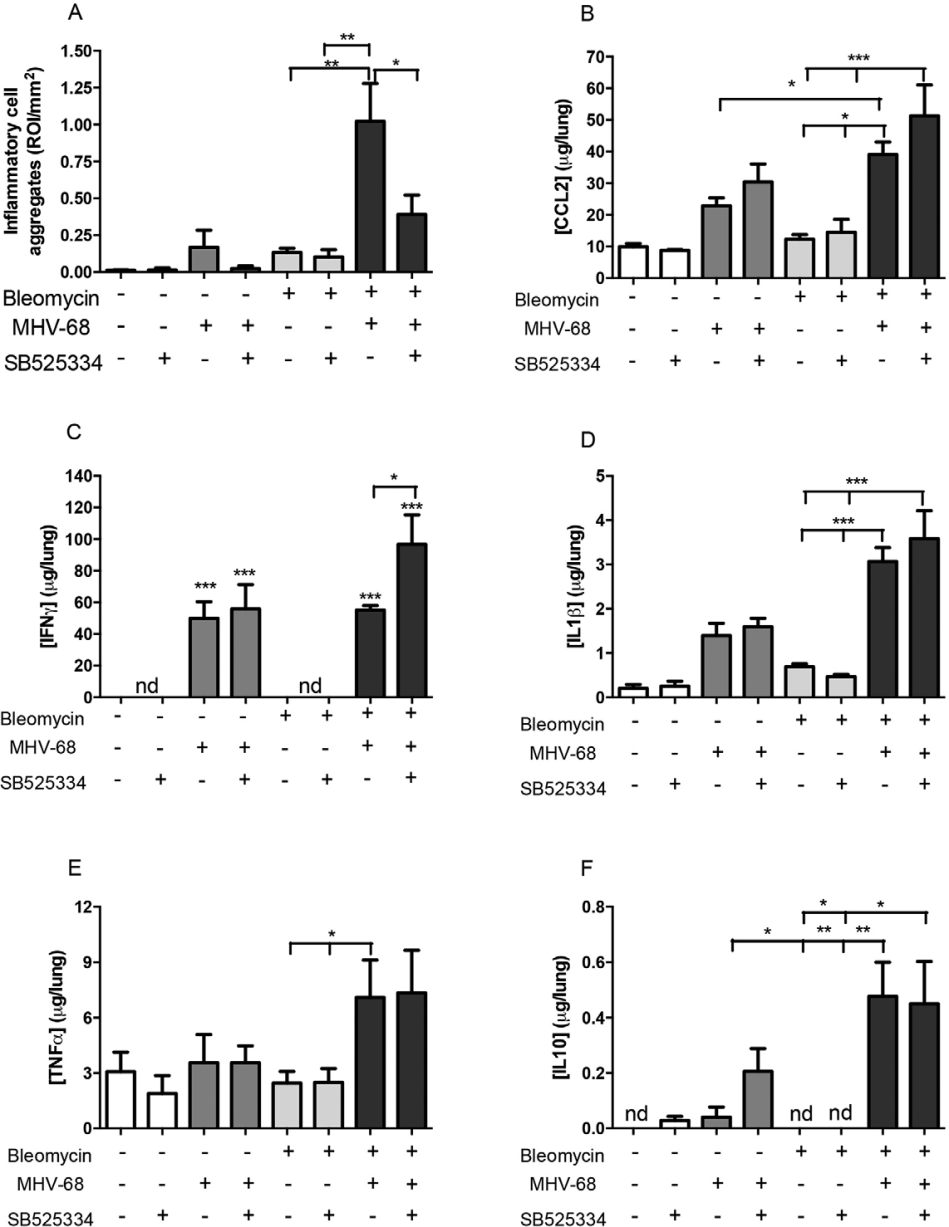
**ALK5 inhibition attenuates inflammatory cell aggregates and enhances IFNγ levels in the two-hit model of MHV-68 infection on a background of pulmonary fibrosis.** (A) Inflammatory aggregates (IAs) were significantly increased in bleomycin- and MHV-68-injured lungs when compared to other bleomycin-challenged groups (*n*=5). SB525334 treatment reduced the number of IAs, quantified and expressed as region of interest per lung (ROI/mm^2^, mean±s.e.m., five tissue sections per mouse). Levels of inflammatory and immunomodulatory markers were measured in lung homogenates: CCL2 (B), IFNγ (C), IL-1β (D), TNFα (E), IL-10 (F); representative of mean±s.e.m., *n*=3 for saline groups and *n*=8 for bleomycin groups. One-way ANOVA, *P<0.05, **P<0.01, ***P<0.001.

The lung abnormalities mapped by µCT analysis were subsequently matched to fibrotic and inflammatory changes identified on hematoxylin and eosin (H&E) and Martius Scarlet Blue (MSB)-stained tissue sections (Fig. 4). Histological analysis confirmed dense patchy fibrosis and collagen deposition in Bleo-injured lungs, which was reduced in mice treated with SB525334. Bleo+MHV-68 lungs displayed evidence of extensive fibrotic lesions and, notably, infiltrations of mononuclear inflammatory cells that formed dense aggregates. We subsequently quantified these inflammatory cell aggregates (IAs) and found that, consistent with our radiological findings, there was little evidence of IAs in Sal+MHV68 lungs. In stark contrast, there were numerous IAs present in the Bleo+MHV-68 two-hit group and these IAs were significantly increased compared with all other experimental groups (Fig. 5A). The number of these IAs was significantly reduced in the two-hit group treated with SB525334 (mean±s.e.m. of Bleo+MHV-68 vs Bleo+MHV-68+SB525334, 1±0.25 vs 0.4±0.13 ROI/mm^2^, *P*<0.05).

We next measured levels of immunomodulatory mediators in lung homogenates and report that viral infection in fibrotic lungs (Bleo+MHV-68) led to a significant increase in lung levels of CCL2 (Fig. 5B), IL-1β (Fig. 5D), TNFα (Fig. 5E) and IL-10 (Fig. 5F) above the levels detected for Bleo lungs. Treatment with SB525334 did not affect the levels of any of these mediators. In contrast, IFNγ was only detectable in virally-infected lungs and IFNγ levels were significantly increased in the Bleo+MHV68+SB525334 group compared to the Bleo+MHV-68 group (mean±s.e.m. of Bleo+MHV-68 vs Bleo+MHV-68+SB525334, 55.14±2.7 vs 96.7±18.6 μg/lung, *P*<0.05) (Fig. 5C).

### Determination of viral gene expression in the two-hit model

**Fig. 6.**
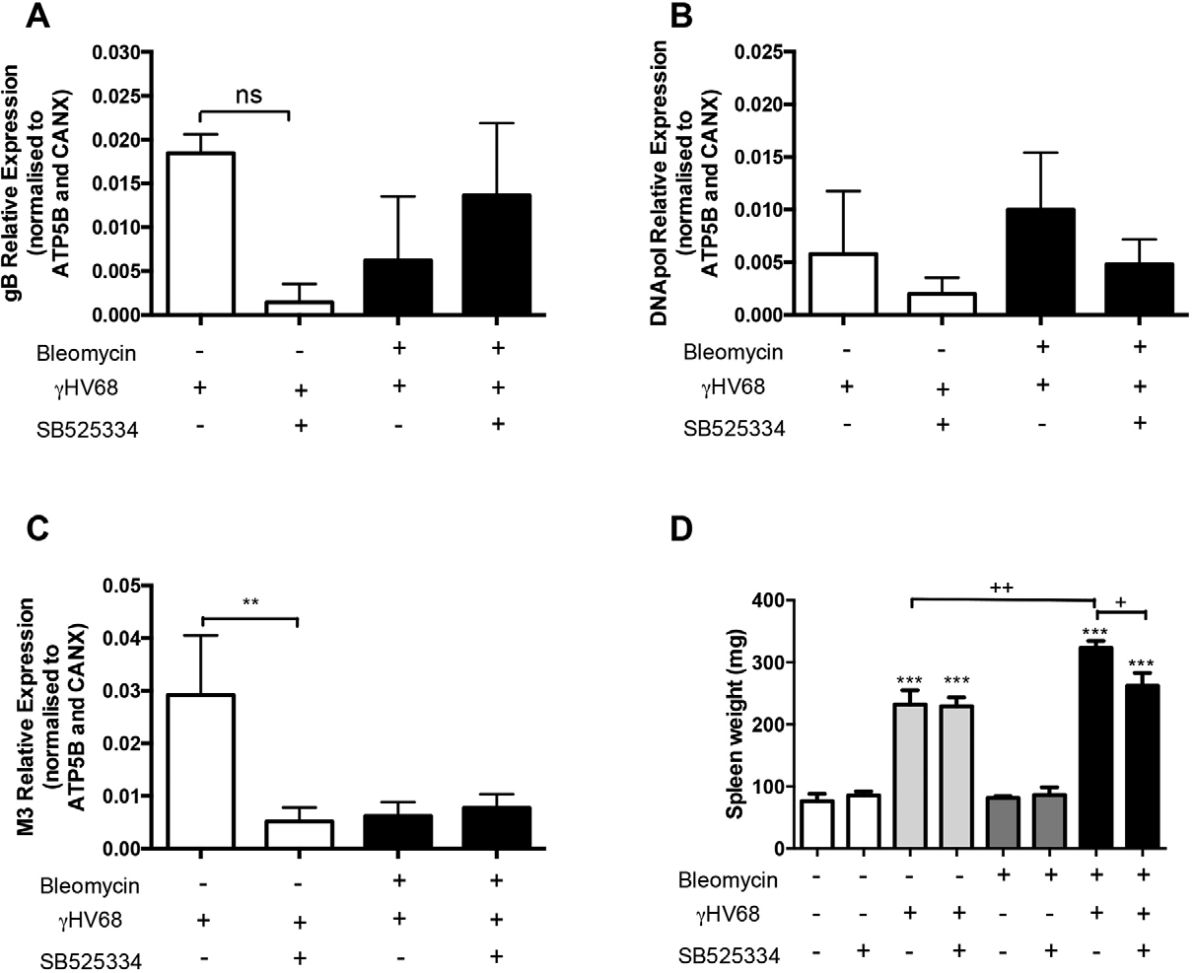
**Detection of viral genes in the lung and splenomegaly indicate ongoing MHV-68 infection.** Viral gene expression was detected in lung tissue 14 days post infection. The viral genes measured included (A) *gB*, (B) *DNApol* and (C) *M3*. Data are representative of mean±s.e.m., *n*=3 for saline groups and *n*=8 for bleomycin groups. One-way ANOVA, **P<0.01. (D) Splenomegaly is detected in all virally-infected groups. Data are representative of mean±s.e.m., *n*=5 for saline groups and *n*=13 for bleomycin groups. One-way ANOVA, ***P<0.001 compared to non-infected groups; ^+^*P*<0.05, ^++^*P*<0.01 comparison within virally-infected groups.

It has been previously reported that active viral replication is required for exacerbation of experimental pulmonary fibrosis (Ashley et al., 2014; McMillan et al., 2008). We therefore evaluated the expression of three viral genes encoding the MHV-68 DNA polymerase and the viral envelope proteins glycoprotein B and M3. In accordance with previous studies (Vannella et al., 2010; McMillan et al., 2008), we show that the viral genes are readily detected in whole lung tissue at the peak of lytic infection 7 days post-infection (p.i.) (supplementary material Fig. 2A-C). By 14 days p.i. the MHV-68 infection had entered a latent phase as demonstrated by decreased levels of viral gene expression in the lung. Blocking TGFβ signalling with the ALK5 inhibitor SB525334 had no impact on viral load in the fibrotic lungs (Fig. 6A-C).

Splenomegaly, a reliable surrogate indicator of herpes virus latent infection (Nash et al., 2001), was evident 14 days p.i. (supplementary material Fig. S2D). In all virally-infected mouse groups, spleen weight was significantly increased when compared to the saline- or bleomycin-only control groups (Fig. 6D). Moreover, splenomegaly was further increased in the Bleo+MHV68 double-hit group compared to single-hit groups, whereas SB525334 treatment significantly reduced spleen weights.

## DISCUSSION

This study aimed to investigate the effect of blocking TGFβ-ALK5 signalling on the progression of lung fibrosis in the presence of concurrent viral infection. We report that MHV-68 viral infection of the fibrotic lung did not increase lung collagen deposition above the levels observed in the bleomycin-alone injured mice, but instead led to the marked accumulation of inflammatory cells, which persisted for at least 14 days p.i. In contrast, saline control lungs inoculated with MHV-68 showed normal architecture. The potent and highly selective TGFβ-ALK5 inhibitor SB525334 (Grygielko et al., 2005) attenuated fibrosis in the single-hit bleomycin model. In contrast, TGFβ-ALK5 inhibition did not significantly block collagen accumulation in the two-hit model but led to a marked reduction in inflammatory cell infiltrates and an enhanced the anti-viral cytokine response. Taken together, these data show for the first time that the therapeutic effect of TGFβ-ALK5 inhibition in lung fibrosis is curtailed in the presence of concurrent viral infection.

### µCT analysis reveals key differences in morphological features between the single- and two-hit models, and the therapeutic effect of ALK5 inhibition

The traditional collagen endpoint measurement used to evaluate fibrosis, based on total lung hydroxyproline levels, has a relatively limited signal window and does not provide information regarding the specific spatial distribution of fibrotic lesions or other potential pathophysiological changes in the injured lung. To complement our biochemical analysis of lung collagen accumulation, we employed *ex vivo* μCT to further characterize the pathological changes in the fibrotic lung with and without concomitant viral infection. As well as providing information regarding the spatial distribution of fibrotic lesions, whole-lung µCT scanning avoids the potential sampling error associated with standard histological analysis of tissue sections. This technology has successfully been applied to the *ex vivo* investigation of lung architecture (Thiesse et al., 2010; Vasilescu et al., 2012) and, more recently, as an endpoint for evaluating fibrosis in single-hit models of fibrosis, based on either bleomycin or adenoviral overexpression of TGFβ (Rodt et al., 2010; Scotton et al., 2013). In agreement with previous data from our laboratory (Scotton et al., 2013), µCT analysis accurately differentiated dense fibrotic lesions associated with bleomycin injury from normal lung morphology. µCT analysis of virally-infected fibrotic lungs revealed diffuse ground-glass opacities and dense consolidation radiating from the bronchovascular bundles, in addition to bleomycin-induced fibrotic lesions. These radiological features are highly reminiscent of those reported in patients with AE-IPF (Collard et al., 2007).

Matching µCT analysis with the histological analysis of the same lungs confirmed that high-density areas corresponded to fibrotic tissue and collagen deposition. The dispersed inflammatory changes evident on µCT scans of virally-infected fibrotic lungs were associated with mononuclear cell aggregates localized around the airways and vasculature. Subsequently, the use of InForm pattern-recognition software was validated in this two-hit model to quantify the changes observed throughout the µCT scans of the whole lungs. It was noted that InForm software accurately highlighted fibrotic lesions in Bleo lungs, whereas, in the two-hit model, inflammation was found to extensively overlap with fibrosis and hence the ‘abnormal lung’ fraction encompassed both types of changes in this model. The µCT analysis confirmed the collagen biochemical data and led us to conclude that, although ALK5 inhibition was effective in preventing fibrotic progression in the single-hit model, this therapeutic effect was attenuated in the two-hit model. Density distribution analysis led us to further postulate that SB525334 primarily targeted inflammatory cell infiltration (lower density voxels) rather than the fibrotic (high density voxels) response in the two-hit model.

In our study we did not demonstrate an increase in lung collagen accumulation following MHV-68 infection on the background of pre-existing fibrosis. This is not a universal finding, and others have reported exacerbation of fibrosis by MHV-68 in the context of FITC and bleomycin models of lung fibrosis (Ashley et al., 2014; McMillan et al., 2008). There could be several potential explanations, including intrinsic differences between the initiating fibrogenic insults as well as their route of administration. FITC is a fine particle that is deposited in the lung and leads to focal chronic inflammation and fibrosis (Moore and Hogaboam, 2008). In contrast, bleomycin causes initial epithelial injury by direct DNA damage and oxidative stress, which in turn triggers a robust inflammatory response leading to inflammatory cell recruitment and vascular leak (Degryse and Lawson, 2011). Increased TGFβ activity by day-14 post-injury is associated with the development of extensive patchy fibrosis (Degryse and Lawson, 2011). The route of administration of bleomycin is known to influence the spatial distribution and evolution of fibrotic lesions, with intratracheal administration resulting in localized bronchiocentric lesions and oropharyngeal administration, as used in our study, causing diffuse peripheral, subpleural lesions (Scotton and Chambers, 2010). Furthermore, whereas the intratracheal model of bleomycin-induced fibrosis resolves over time (Degryse et al., 2010), recent evidence from our laboratory demonstrated that the oropharyngeal mode of bleomycin instillation leads to persistent fibrosis and collagen deposition with little evidence of restoration of lung architecture up to at least 6 months post-injury (Scotton et al., 2013). These key differences between models might be crucial in terms of determining the subsequent effect of MHV-68 infection on the progression of the fibrotic response. In addition, we addressed the possibility that the differences between the reported studies could have arisen from using different methods of collagen quantification. In agreement with our HPLC data, standard Sircol colorimetric assay confirmed the lack of exacerbated lung collagen accumulation in our model. In contrast, all studies agree that MHV-68 infection triggered a robust and persistent inflammatory response on a background of pre-existing fibrosis.

### ALK5 inhibition targets inflammatory cell infiltration in the two-hit model

MHV-68 infection alone leads to the long-term release of immunomodulatory mediators by resident and recruited cells in the lung, including: TNFα by mesenchymal cells, B cells and alveolar macrophages; CCL2 and IFNγ by alveolar macrophages; and IFNγ and IL-10 by T cells (Sarawar et al., 1996; Stoolman et al., 2010). Our two-hit model clearly demonstrates that, in the event of infection concomitant with pre-existing fibrosis, the inflammatory response is exacerbated, persistent and associated with a further increase in the accumulation of mediators that might also perpetuate the profibrotic milieu. CCL2 is readily detected in the sera and bronchoalveolar lavage fluid of IPF patients (Baran et al., 2007; Suga et al., 1999) and promotes fibrocyte and inflammatory cell recruitment (Moore et al., 2005) as well as collagen production by fibroblasts (Kim et al., 2014). In models of MHV-68-mediated exacerbation of FITC-induced fibrosis (McMillan et al., 2008) and fibrosis in latently infected lungs (Vannella et al., 2010), high levels of CCL2 and CCL12 are detected in virally-infected fibrotic lungs. Overexpression of IL-1β *in vivo* leads to acute alveolar and parenchymal inflammation that progresses into interstitial fibrosis with accumulation of fibroblasts and myofibroblasts in the lung (Kolb et al., 2001). Similarly, overexpression of TNFα in rat lungs leads to acute inflammation and fibrosis (Sime et al., 1998). Overexpression of IL-10 has also been linked to the development of pulmonary fibrosis *in vivo* (Sun et al., 2011). Although current evidence suggests that TGFβ is an important driver of lung collagen accumulation during days 14 to 28 post-bleomycin in the single-hit model (Scotton et al., 2013), it is plausible that, in the two-hit model, the TGFβ-independent, additive profibrotic actions of these mediators perpetuate ECM deposition and hence override the antifibrotic effect of SB525334.

In contrast to the lack of therapeutic effect of ALK5 inhibition on lung collagen accumulation in the two-hit model, the Bleo+MHV-68 infected lungs harboured the highest number of inflammatory aggregates (IAs) compared with all other experimental groups, and this parameter was reduced in response to ALK5 inhibitor treatment. TGFβ plays a key immunomodulatory role, including inhibition of CD4 T-cell differentiation, IFNγ production, induction of regulatory T cells and inhibition of antigen-presenting-cell function (Odeberg and Söderberg-Nauclér, 2001). In models of Herpes simplex virus-1 (HSV-1) infection, inhibition of TGFβ signalling in immune cells leads to the expansion of natural killer (NK) cells, increased IFNγ production and hence better control of viral infection, which in turn is associated with reduced immune cell infiltration at the site of infection (Allen et al., 2011). Moreover, inhibition of TGFβ signalling decreases the viral capacity to establish latency and reduces the number of immune cells infiltrations into the primary site of infection as well as to the site of latency (Allen et al., 2011). In bone-marrow-transplantation models, latent MHV-68 infection leads to chronic and persistent pneumonitis and fibrosis, which is associated with the accumulation of macrophages and the influx of neutrophils and lymphocytes into the lung, with the latter being dominated by CD8 and CD4 T cells (Coomes et al., 2011). Importantly, blocking TGFβ signalling in T cells leads to an enhanced antiviral responses and attenuation of inflammation and fibrosis (Coomes et al., 2010, 2011). Consequently in our two-hit model, ALK5 inhibition reduced the number of IAs in the lung and attenuated splenomegaly. Interestingly, these responses were not associated with any reduction in viral gene expression in the lung; rather, they were associated with increased levels of IFNγ in the virally-infected fibrotic lungs treated with the TGFβ-ALK5 inhibitor. IFNγ does not play a direct role in viral clearance from the lung (Dutia et al., 1997) but it is a key cytokine involved in antiviral immunity, essential for CD8 T-cell-mediated responses during acute lytic infection and for CD4 T-cell-dependent control of persistent infection (Christensen et al., 1999). IFNγ-receptor-deficient mice show increased perivascular accumulations of immune cells, primarily B cells, in response to MHV-68 infection (Lee et al., 2009). It is plausible that the increase in IFNγ levels is associated with the decrease in IAs observed in our study. This might actually be beneficial to the host, because viral load and disease severity are often neither linear nor indeed associated. The immunopathology associated with the viral infection is often more damaging than the virus itself, and, in the case of γ-herpesviruses, this immunopathology is associated with Th1-type cytokine expression, inflammation and bystander tissue damage (Nash et al., 2001). Antiviral therapies have been shown to be beneficial in a subset of IPF patients with evidence of EBV infection (Egan et al., 2011), and attenuate fibrosis resulting from chronic MHV-68 infection in animal models (Mora et al., 2007). Our results point to an interesting prospect of combining anti-TGFβ and antiviral therapies as a potential treatment for pulmonary fibrosis.

Furthermore, the balance between the lytic and latent phases of infection is also likely to influence the progression of fibrosis. In our studies we confirmed the switch from lytic phase (7 days p.i.) to the latent phase (14 days p.i.) by measuring the spleen weights and viral gene expression in the lungs. To the best of our knowledge, this is the first report evaluating the progression of fibrosis in the two-hit model at 14 days p.i., which could be another explanation for the lack of exacerbation of collagen deposition. Previous studies (Ashley et al., 2014; McMillan et al., 2008) measured collagen deposition at 7 days p.i., at the height of the extremely cytotoxic and inflammatory lytic phase. Another study reported that latent MHV-68 infection induced a pro-fibrotic phenotype in lung tissue (Stoolman et al., 2010) and showed exacerbated fibrotic responses in the latently infected lungs (Vannella et al., 2010). This highlights the need for further longitudinal studies to assess the relative contributions of herpesvirus viral replication, latency and specific host responses on disease severity and concomitant pulmonary fibrosis.

### Conclusions and implications

In conclusion, we report that MHV-68 infection on the background of pre-existing fibrosis leads to a robust and persistent inflammatory response that is reminiscent of ground glass opacities and consolidation reported in patients with AE-IPF. Targeting TGFβ-ALK5 signalling in the fibrotic lung prevents further progression of fibrosis in the single-hit model but this effect is attenuated in the presence of concurrent viral infection. In contrast, inhibiting TGFβ-ALK5 signalling in this context increases the levels of the antiviral cytokine IFNγ and reduces inflammatory cell infiltration. These observations highlight the importance of the pleiotropic nature of TGFβ-ALK5 signalling in immune and antiviral responses in determining the anti-fibrotic effect of TGFβ-ALK5 inhibition in the presence of viral infection. These findings have potential important therapeutic implications in terms of targeting this signalling axis in human fibrotic lung disease.

## MATERIALS AND METHODS

### MHV-68 infection on the background of pulmonary fibrosis

All studies were ethically reviewed and performed in accordance with the UK Home Office Animals for Scientific Procedures Act 1986. C57BL/6 male mice between 10 and 12 weeks of age (Charles River Laboratories, UK) were administered bleomycin (25 IU/mouse in 50 μl of sterile 0.9% saline) or saline by oropharyngeal instillation as previously described (Lakatos et al., 2006).

Two weeks after bleomycin instillation, mice were anaesthetised by intraperitoneal injection of ketamine (80 mg/kg body weight) and xylazine (8 mg/kg body weight). 1×10^5^ plaque forming units (PFUs) of MHV-68 (ATCC, Manassas, VA, USA) suspended in 20 μl sterile saline were inoculated intranasally. Mice were sacrificed 7 or 14 days p.i. by intraperitoneal injection of pentobarbitone and severing of the abdominal inferior vena cava.

### ALK5-inhibitor study

The highly selective ALK5 inhibitor SB525334 (Grygielko et al., 2005) was a kind gift from Novartis, Horsham, UK. The compound (30 mg/kg body weight in 100 μl acidified saline/0.2% Tween 80 pH 4.1) or vehicle (acidified saline/0.2% Tween 80 pH 4.1) was administered from day 15 post-bleomycin-instillation, which corresponds to 1 day p.i., twice daily by oral gavage for the remaining duration of the experiment. Treatment combinations are summarized in Table 1.

**Table 1.**
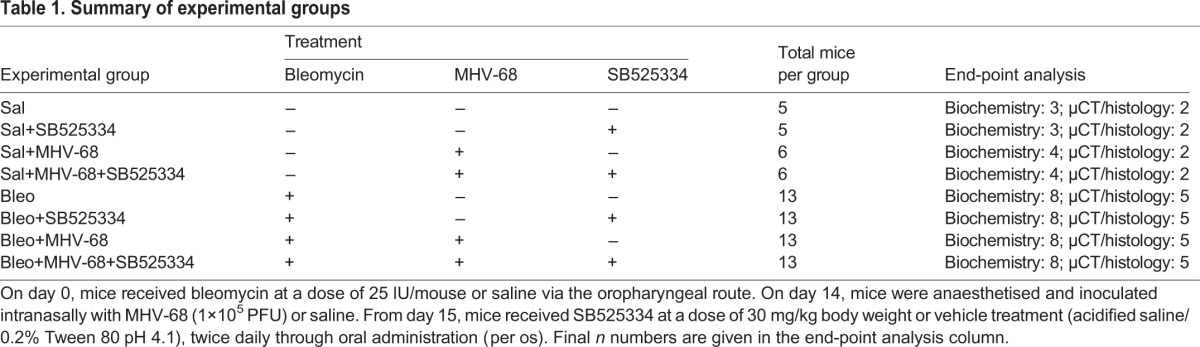
**Summary of experimental groups**

For measurements of total collagen and inflammatory mediators, the lungs were snap-frozen in liquid nitrogen, weighed and pulverized to homogeneity. For μCT, histological and immunohistochemical analysis, the lungs were insufflated with 4% paraformaldehyde at a constant pressure of 20 cm H_2_O, fixed for 24 h then stored in 70% ethanol. Spleens were snap-frozen in liquid nitrogen and weighed.

### Determination of total lung collagen

Total lung collagen was calculated by measuring hydroxyproline content in aliquots of pulverized lung. Hydroxyproline was quantified by reverse-phase HPLC of NBD-Cl-derived acid hydrolysates of the pulverized lung and the value used to calculate total lung collagen based on the average hydroxyproline content of collagen (12.2%).

Total lung collagen was also measured using the Sircol assay (Biocolor Ltd, UK) according to the manufacturer's instructions. Briefly, a small quantity of pulverized lung was accurately weighed and acid-pepsin extracted. The quantity of collagen was calculated in mg per lung.

### Micro-computed tomography (μCT) imaging

Insufflated lungs were incubated for 2 h each in increasing concentrations of ethanol (70%, 80%, 90%), then 100% ethanol overnight before being transferred to 100% hexamethyldisilazane for another 2 h and then air-dried. Lungs were scanned in a SkyScan 1072 μCT scanner (SkyScan, Kontich, Belgium) at 40 kV/100 μA, without a filter, using two frame averaging at a 0.49° angular rotation step size, and a voxel size set to 12.8 µm with typical Modulation Transfer Function (MTF) of around 10%; spatial resolution was in the region of 20-30 μm. Scan time was around 10 min, allowing high-resolution visualization and good throughput. Reconstruction was carried out with the SkyScan NRecon software (SkyScan, Kontich, Belgium). Also see Scotton et al. (2013) for further details.

### μCT image analysis

Tissue segmentation analysis was performed using InForm™ software (PerkinElmer, UK) as previously described (Scotton et al., 2013). The software training algorithm was set to discriminate between normal versus abnormal lung and gate out any non-lung tissue as tested on three representative μCT sections (8-bit greyscale) from each animal in the study until over 90% accuracy was achieved. Saline control and Bleo+MHV-68 lungs were set as a standard for normal and abnormal lung categories, respectively. All μCT sections (∼900 sections per lung) were subsequently segmented using the same algorithm on a medium sample area at fine resolution. The output measurements were pixel area and pixel density for each category that were then compiled into a composite measurement of abnormal lung volume expressed as a percentage of total lung volume, and greyscale density expressed as total lung density.

### Voxel density distribution analysis

Frequency distribution of voxel densities in unsegmented lung was analyzed by generating composite 256-colour greyscale histograms (from 0=black to 255=white). The mean number of voxels in each bin (1 greyscale unit wide) was calculated along with statistical analysis of differences between the experimental groups.

### Histology

Paraformaldehyde-fixed lungs were dehydrated and embedded in paraffin wax blocks. For standard histological processing, 3-5 μm paraffin sections were mounted on polylysine-coated glass slides and dewaxed. Hematoxylin and eosin (H&E) and modified trichrome (Martius Scarlet Blue [MSB]) staining was performed using an automated Sakura Tissue-Tek DRS 2000 Multiple Slide Stainer. All sections were subsequently scanned on a Nanozoomer and images were captured using NDP.view v.1.2.36 (both from Hamamatsu Corporation, Hamamatsu, Japan).

Direct comparisons between μCT and histology were performed on the same set of lungs: post-μCT lungs were rehydrated through an ethanol gradient (100%, 90%, 80% and 70% for 2 h in each) prior to standard processing as above.

### Inflammatory cell aggregate (IA) counts

IAs were identified and quantified in H&E sections using Nuance^®^ FX Multispectral Tissue Imaging Software (PerkinElmer, UK). The software unmixed and enhanced the areas of intense haematoxylin staining that corresponded to IAs and quantified them as regions of interest per area of the lung section (ROI/mm^2^; five whole histological sections per animal).

### Measurements of inflammatory markers

Lung powders were homogenized in PBS/1% Triton X (Sigma, UK)/proteinase inhibitor cocktail (Roche, UK) using a freeze-thaw cycle. CCL2 DuoSet ELISA Development kits were purchased from R&D Systems, USA, and used according to the manufacturer's instructions. The optical density was measured using a plate reader (Multiskan MCC/340, Titertek) at dual wavelength A1: 450 nm and A2: 540 nm. Mouse pro-inflammatory Panel 1 V-Plex Plus Kit was purchased from Meso Scale Discovery (Rockville, MD, USA) for quantification of the following ten cytokines that are important in infection and inflammation: IFN-γ, IL-1β, IL-2, IL-4, IL-5, IL-6, KC/GRO, IL-10, IL-12p70 and TNF-α. The kit was used according to manufacturer's protocol. Sector Imager 600 MSD plate reader and MSD Discovery Workbench software were used to record and analyze the results.

### RT-PCR for viral mRNA

Total RNA from frozen powdered lung tissue was isolated with TRIzol reagent as per the manufacturer's protocol (Invitrogen). RNA was DNase-treated using a DNAfree kit (Ambion). Real-time RT-PCR was conducted using the Platinum SYBR Green qPCR SuperMix UDG (Invitrogen, UK) with cycling conditions as follows: 1 cycle of 50°C for 2 min and 95°C for 2 min; 45 cycles of 95°C for 5 s, 55°C for 5 s and 72°C for 15 s. The specificity of the PCR product was confirmed by melting-curve analysis. The gB, DNApol and M3 primer sequences were previously published (McMillan et al., 2008). For each gene, crossing point (Cp) values were determined from the linear region of the amplification plot and normalized by subtraction of the geometric mean of the crossing point (Cp) values for two housekeeping genes: ATP synthase 5B (*ATP5B*) and calnexin (CANX), identified by GeNorm analysis as the most stable housekeeping genes for this study. Relative expression was subsequently calculated using the 2-ΔCp approach. All primers and GeNorm kits were purchased from Primer Design (Southampton, UK).

### Statistical analysis

All data are presented as mean values±s.e.m., unless indicated otherwise. Statistical analysis was performed between two treatment groups by Student's *t*-test, and between multiple treatment groups by one-way analysis of variance (ANOVA), using Graphpad Prism 5 software. A *P*-value of <0.05 was considered significant.

## Supplementary Material

Supplementary Material
