# Supplementary material for: The anti-fibrotic effect of inhibition of TGFβ-ALK5 signalling in experimental pulmonary fibrosis in mice is attenuated in the presence of concurrent γ-herpesvirus infection
Source: Dis Model Mech. 2015 Sep 1;8(9):1129–39. doi: 10.1242/dmm.019984 (PMC4582104; doi:10.1242/dmm.019984)
Supplement: Supplementary Material [file supp_8_9_1129__index.html]

Supplementary Material 

# The anti-fibrotic effect of inhibition of TGFβ-ALK5 signalling in experimental pulmonary fibrosis in mice is attenuated in the presence of concurrent γ-herpesvirus infection

## DMM019984 Supplementary Material

- Supplementary Material
