# Supplementary material for: The anti-fibrotic effect of inhibition of TGFβ-ALK5 signalling in experimental pulmonary fibrosis in mice is attenuated in the presence of concurrent γ-herpesvirus infection
Source: Dis Model Mech. 2015 Sep 1;8(9):1129–39. doi: 10.1242/dmm.019984 (PMC4582104; doi:10.1242/dmm.019984)
Supplement: Supplementary Material [file supp_019984_DMM019984supp.pdf]

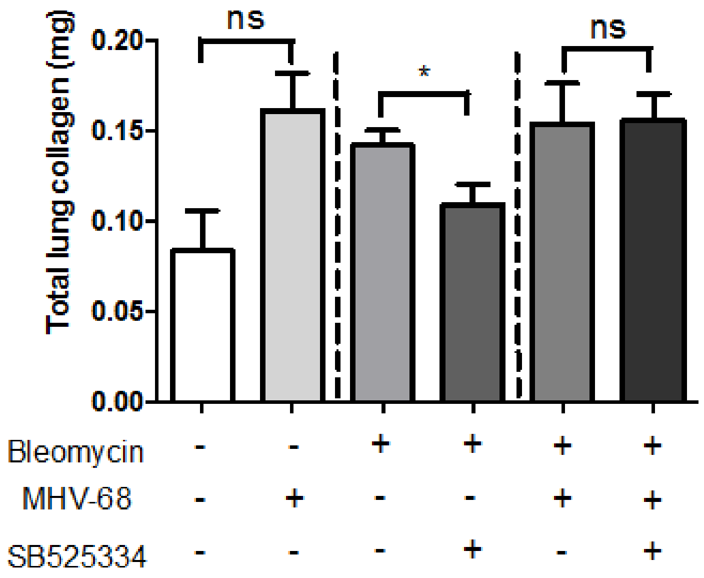

Figure S1. The anti-fibrotic effect of TGFβ/ALK5 signalling inhibition is attenuated in the two-hit model of MHV-68 infection on the background of pre-existing fibrosis.

Lung collagen quantification by Sircol assay 28 days post-oropharyngeal bleomycin instillation (corresponding to 14 days p.i. with MHV-68) showed a broadly similar pattern to that determined by HPLC. Data are representative of mean +/- SEM, n=3 for saline groups and n=8 for bleomycin groups; statistical analysis, Student t-test, *$p<0.05$.

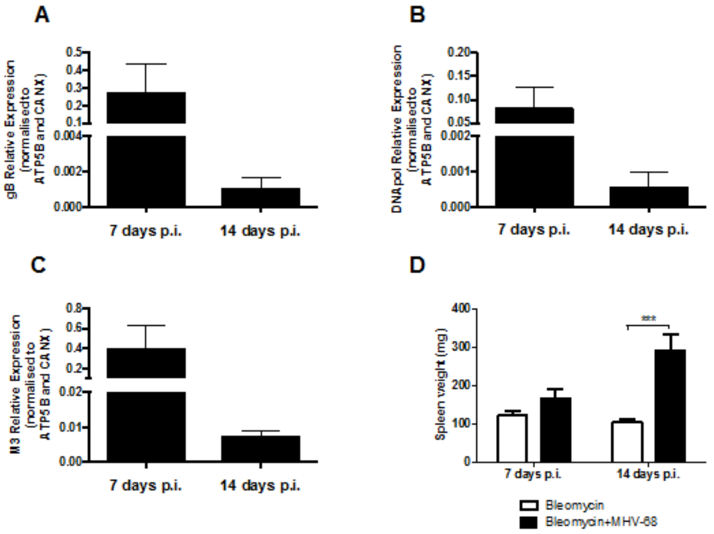

Figure S2. MHV-68 infection switches from lytic to latent phase between 7 and 14 days post infection.

The levels of viral gene expression in the lung were detected at 7 and 14 days p.i. and included **(A)** gB, **(B)** DNApol and **(C)** M3. Data are representative of mean +/- SEM, n=4-5 animals per group. **(D)** Splenomegaly was also measured 7 and 14 days p.i. Data are representative of mean +/- SEM, n=7-9 animals per group. One-way ANOVA, ***p<0.001.
